# Effects of an Early Literacy Intervention for Linguistically Diverse Children: A Quasi-Experimental Study

**DOI:** 10.3389/fpsyg.2020.569854

**Published:** 2020-09-29

**Authors:** Pascale M. J. Engel de Abreu, Silke Fricke, Cyril Wealer

**Affiliations:** ^1^Institute for Research on Multilingualism, University of Luxembourg, Esch-sur-Alzette, Luxembourg; ^2^Division of Human Communication Sciences, The University of Sheffield, Sheffield, United Kingdom

**Keywords:** intervention, kindergarten, literacy, foundational skills, phonological awareness, letter-knowledge, linguistically diverse

## Abstract

Phonological awareness and letter-sound knowledge underpin children’s early literacy acquisition. Promoting these foundational skills in kindergarten should therefore lead to a better response to formal literacy instruction once started. The present study evaluated a 12-week early literacy intervention for linguistically diverse children who are learning to read in German. The study was set in Luxembourg where kindergarten education is in Luxembourgish and children learn to read in German in Grade 1 of primary school. One hundred and eighty-nine children (mean age = 5;8 years) were assigned to an early literacy intervention in Luxembourgish or to a business as usual control group. Trained teachers delivered the intervention to entire classes, four times a week, during the last year of kindergarten. The early literacy program included direct instruction in phonological awareness and letter-knowledge, while promoting print and book awareness and literacy engagement. Children were assessed pre-intervention, immediately post-intervention and at a 9 months delayed follow-up using measures in Luxembourgish and in German. At the end of the intervention, children in the intervention group performed significantly better than the control group on phonological awareness and letter-knowledge measures in Luxembourgish and the gains in phonological awareness were maintained at 9 months follow-up. The effects generalized to measures of phonological awareness, word-level reading comprehension and spelling in German (effect sizes *d* > 0.25), but not to German single word/pseudoword reading, at delayed follow-up. Intervention programs designed to support foundational literacy skills can be successfully implemented by regular teachers in a play-based kindergarten context. The findings suggest that early literacy intervention before school entry can produce educationally meaningful effects in linguistically diverse learners.

## Introduction

Being able to read and write are among the most important academic skills and key to educational success. However, literacy acquisition can be difficult, and many students struggle for a variety of reasons. Children with limited proficiency in the language of literacy instruction often experience particular challenges in the acquisition of literacy skills, which has been globally acknowledged as a pressing concern (Snow et al., 1998; August and Shanahan, 2006; Weber et al., 2007). Evidence-based teaching approaches to support at-risk children can make a significant difference in later educational outcomes (Snowling and Hulme, 2011). The efficacy and effectiveness of systematic and explicit phonological awareness training combined with letter-knowledge instruction has been supported by rigorous evidence, which has led to the implementation of these evidence-based practices in kindergarten curriculums in many countries (Bus and van IJzendoorn, 1999; National Institute of Child Health and Human Development, 2000; Ehri et al., 2001; Rose, 2006; Kelly et al., 2019). Nonetheless, questions remain about how such interventions can be most effectively delivered in different educational contexts and across a variety of learners. Furthermore, studies assessing the effects of early literacy interventions in children who acquire literacy in languages other than English and in children who learn to read in a second language are scarce (August and Shanahan, 2006; Weber et al., 2007; Murphy and Unthiah, 2015; Oxley and De Cat, 2019).

This study explored the effects of a kindergarten intervention in Luxembourgish on early literacy skills in German in children from Luxembourg where the language of instruction is Luxembourgish in kindergarten and all children learn to read in German in Grade 1 of primary school. Luxembourgish is a West Germanic language spoken in Luxembourg, that has structural similarities (e.g., cognate vocabulary or phonological forms) with Standard German (Gilles and Trouvain, 2013). We developed a new program in Luxembourgish that draws on previous studies suggesting a causal link between phonological awareness and letter-knowledge as a foundation for early literacy skills (Melby-Lervåg et al., 2012). Accordingly, we trained those foundational skills in the context of a literacy enrichment program. Central to the study is the question whether an early literacy intervention in Luxembourgish can successfully support literacy acquisition in German in linguistically diverse learners.

### Theoretical Framework

The Simple View of Reading (Gough and Tunmer, 1986) proposes that to become successful readers, children need to be able to decode written text and understand language, which is crucial for reading for meaning. According to this theoretical framework, decoding is a critical step in the process of becoming literate and limitations in decoding impede reading comprehension (Castles et al., 2018). Strong decoding skills, on the other hand, have been shown to enable advanced levels of written text processing (Cain and Oakhill, 1999).

To develop robust decoding skills, children need to establish a secure linkage between letters and the sounds of a language (Hatcher et al., 1994, 2004). To do this, they need to understand how letters in printed words translate into phonemes in spoken words (i.e., grapheme-to-phoneme correspondences). It is now widely accepted that there is a strong relationship between reading and spelling, and children’s phonological awareness and their letter-knowledge skills across a range of orthographies (see Melby-Lervåg et al., 2012 for a review). In a study with monolingual children from Germany, letter-knowledge and phonological awareness were identified as the two most important school-entry predictors of literacy development in German (Fricke et al., 2008). Similar results were obtained in a longitudinal study from Luxembourg, in which phonological awareness in Luxembourgish emerged as the most important predictor of success in learning to read in German (Engel de Abreu and Gathercole, 2012). The development of phonological awareness is thought to progress from larger (i.e., syllables and rimes) to smaller linguistic units (i.e., phonemes). While syllable and onset-rime awareness develop more naturally to a reliably measurable degree in preschool children, phoneme awareness is generally not yet well developed (Carroll et al., 2003). There exists a consensus that across orthographies, phoneme-level skills tend to be a better predictor of children’s literacy progress than phonological awareness skills at larger linguistic unit size at early stages of literacy acquisition (Caravolas et al., 2005).

Alphabetic writing systems differ in terms of their consistency of grapheme-to-phoneme and phoneme-to-grapheme correspondences. German is an orthography with relatively consistent grapheme-to-phoneme correspondences involved in reading (Landerl and Wimmer, 2008). Children generally acquire letter-knowledge and word-level decoding skills relatively fast in German as opposed to less consistent orthographies such as English for example (Aro and Wimmer, 2003). In contrast to the relatively consistent grapheme-to-phoneme correspondences, phoneme-to-grapheme correspondences needed for spelling are less consistent in German. Indeed, spelling in German depends to a greater extent on phonological awareness skills than reading (Landerl and Wimmer, 2008; Fricke et al., 2016). A systematic review of longitudinal research in German-speaking countries has shown that phonological awareness was a better predictor of spelling and reading comprehension than of reading speed or accuracy (Pfost, 2015). Similar findings emerged from research with at-risk children. Wimmer et al. (2000) found that children with low phoneme awareness skills at school entry struggled with spelling and irregular word reading, whereas no problems were observed in non-word reading or in regular word reading in German.

The longitudinal predictive relationships suggest that early literacy development critically depends on the cognitive skills tapped by phonological awareness and letter-knowledge. Experimental studies indicate that training these foundational skills is indeed effective in helping to improve children’s early literacy development. In a United Kingdom-based randomized controlled trial, Bowyer-Crane et al. (2008) evaluated an intervention program fostering phoneme awareness and letter-sound knowledge in 4-year-old children with poor oral language proficiency. Results showed that, in comparison to an active oral language control group, children in the early literacy intervention group made significantly more improvements in phoneme awareness, decoding and single word reading. In another intervention study from the United Kingdom, with linguistically diverse children, Stuart (1999, 2004), showed that whole-class phoneme awareness and letter-knowledge teaching in 5-year-olds improved English single word reading, decoding and spelling skills in Grade 1 and Grade 3.

### Effects of Early Literacy Interventions on Learning to Read in German

Research examining the efficacy of early literacy interventions for children who are learning to read in German are less available than for English-speaking countries. In a meta-analyses of the effects of training phonological awareness in German, Fischer and Pfost (2015) found positive intervention effects across 19 studies for phonological awareness (*d* = 0.36), letter-sound knowledge (*d* = 0.26), decoding (*d* = 0.18), reading comprehension (*d* = 0.26) and spelling (*d* = 0.26). In their study, Schneider et al. (1997) explored the efficacy of a phonological awareness training that was administered in regular kindergarten classes in Germany. Results showed that the intervention was essentially only effective if the crucial component of phoneme-level activities was consistently administered. A follow-on study showed that combining the phonological awareness training with instruction in letter-knowledge produced larger effects on subsequent literacy achievement than phonological awareness or letter-knowledge training alone (Schneider et al., 2000). A recent intervention study from Germany that trained phonological awareness and letter-knowledge indicates strong intervention effects on phonological awareness and letter-knowledge skills that did, however, not generalize to reading. Small effects on word reading were only identified in a group of low-performing children (Pfost et al., 2019).

Orthographic consistency has been put forward as a possible factor that might account for observed differences in training effects on literacy skills across different languages. Some have argued that within the relative consistent German orthography, phonological awareness skills are less important for learning to read which could explain why phonological awareness intervention studies produce smaller (if any) significant effects on literacy outcomes in German (Fischer and Pfost, 2015; Wolf et al., 2016; Pfost et al., 2019) than in less consistent orthographies such as English for example. Notably, what most early literacy training studies from Germany have in common is that they explored the effectiveness of interventions that follow the strategies of a specific program (*Hören*, *Lauschen*, *Lernen*, HLL, Plume and Schneider, 2004; Küspert and Schneider, 2008) that is based on a phonological awareness training from Denmark by Lundberg et al. (1988). A meta-analysis by Bus and van IJzendoorn (1999) showed that the Lundberg phonological awareness intervention (that does not include letter-knowledge activities) consistently produced strong effects on phonological awareness skills (*d* = 1.33) but small effects on reading (*d* = 0.15) across different countries including Germany, Denmark, the United Kingdom, and Israel.

While some positive effects have been reported in early literacy intervention studies from Germany, most have explored the same training program (i.e., HLL) that starts with more general listening and language games on phrase and word level, before moving on to phonological awareness activities on syllable, onset-rime and finally phoneme level that may be supplemented with letter-knowledge training. Studies have shown that teachers are likely to omit the crucial phoneme-level activities of the HLL intervention because they either run out of time or are unfamiliar with the structured exercises at the phoneme-level and encounter difficulties when introducing those in their classrooms (Schneider et al., 1997; Bodé and Content, 2011).

### Cross Language Effects of Early Literacy Interventions

It is currently unclear whether training foundational skills in one language can facilitate learning to read in another language (Genesee et al., 2006a; Melby-Lervåg and Lervåg, 2011). According to the linguistic interdependence hypothesis (Cummins, 1979), transfer between languages can occur because different languages rely upon a common underlying proficiency. What the common proficiency actually represents and to which domains of language it applies, however, is less clear (Genesee et al., 2006b). There is evidence to suggest that cross language transfer (or facilitation) occurs for lower-order aspects of linguistic competence, such as phonological awareness and decoding skills. In their meta-analyses, Melby-Lervåg and Lervåg (2011) found moderate to large correlations for those lower order skills across languages and the authors posit this transfer occurs because general strategies can be taught effectively for skills that involve a limited number of sounds and letter-sound combinations. However, studies exploring cross-linguistic transfer of code-related skills show large variations in results (Bialystok et al., 2005; Swanson et al., 2008; Wawire and Kim, 2018). Language typology has been suggested to play a role in cross language transfer effects with transfer being more likely between languages that are similar (e.g., share cognate vocabulary, phonological forms, and writing system) than between languages that share fewer features (Odlin, 1989; Connor, 1996).

In general, there is a dearth of evidence for the transfer of language skills from one language to another and intervention studies exploring cross-linguistic transfer of early literacy skills to languages other than English are almost non-existent. To the best of our knowledge, only one study has explored the effects of a phonological awareness training in Luxembourgish on the development of literacy skill in German. In their study, Bodé and Content (2011) administered a Luxembourgish adapted version of the phonological awareness training (without letter-knowledge instruction) used in studies from Germany (HLL, Schneider et al., 1997) to linguistically diverse children in Luxembourg. For a random sample of kindergarten children, intervention effects were found on a number of phonological awareness tasks in Luxembourgish, but gains did not generalize to letter-knowledge and spelling in German in Grade 1. Significant training effects on Grade 1 German pseudoword spelling skills were only found for a subgroup of children with low kindergarten phonological awareness scores, but those analyses excluded second language learners of Luxembourgish (approximately 37% of the sample).

Taken together, the review of the literature has shown that few early literacy intervention studies include linguistically diverse learners and few explore cross-language effects of interventions. There is, therefore, a clear need for further research on effective instructional approaches to support the literacy development in young children from diverse backgrounds. It has been acknowledged that such approaches should be adapted to the individual needs of children (Stuart, 1999, 2004; Pfost et al., 2019).

### The Current Study

The present study took place in the Grand Duchy of Luxembourg, a small trilingual country in Europe. Luxembourgish is the national language of Luxembourg and one of its three official languages (together with French and German). Luxembourgish is widely used across the entire country. Although Luxembourgish has a fully developed orthography, children in Luxembourg learn to read and write in German, which is typologically close to Luxembourgish (Gilles, 2020). The syllable structure and phonological system of Luxembourgish for example are similar to Standard German and the morpho-syntax follows Germanic patterns (Gilles and Trouvain, 2013). Standard German, besides its role as language of literacy instruction, is mostly used as a passive language of print media and partly as written language of public administration (Gilles, 2020). Luxembourg’s educational system is multilingual. Children start compulsory education at the age of four and the first two years are spent in kindergarten (*Cycle 1*). Kindergarten instruction is in Luxembourgish and does not include formal literacy or letter-knowledge instruction. Teaching is foremost a social and play-based experience. Literacy instruction does not start until the age of six when children enter Grade 1 of primary school. German at this stage is taught as an additional language as well as used, together with Luxembourgish, as the language of instruction. Children learn to read and write in German (not in Luxembourgish). Luxembourg is linguistically and culturally diverse and approximately 47% of the population are foreign-born residents (Institut national de la statistique et des études économiques du Grand-Duché de Luxembourg, 2020). Approximately 59% of all the students speak a language other than Luxembourgish at home and approximately 27% of the students indicate Portuguese as their home language (Lenz and Heinz, 2018).

Building on previous evidence, we developed a new early literacy program in Luxembourgish for kindergarten. Our intervention uses established strategies to foster phonological awareness and letter-knowledge skills embedded in a broad and language-rich program and follows didactic principles that have been shown to be effective when teaching linguistically diverse learners (August et al., 2005; Tonnar et al., 2010; Chumak-Horbatsch, 2012; Richards-Tutor et al., 2016). The intervention ran for 12 weeks and was administered as a whole-class program by the class teacher in the last year of kindergarten when children are 5–6 years old. Phonological awareness and letter-knowledge skills in Luxembourgish were assessed before and immediately after the intervention. We also tested phonological awareness, word/pseudoword reading, word-level reading comprehension skills and spelling in German at delayed follow-up to determine whether intervention effects would transfer to early literacy skills in German. Intervention effects were explored in a random sample of children from public kindergartens in Luxembourg. Given that approximately half of Luxembourg’s student population are Luxembourgish second language learners, an additional interest of the study was to explore the effects of our intervention for children with limited oral language proficiency in the language of kindergarten instruction. Theoretically, this study adds to our understanding of cross-linguistic influence on the early literacy development in multilingual children and educational contexts, which represents an area that has received little research attention (Snow, 2008). Practically, the study can contribute toward the design of early evidence-based support programs for preparing linguistically diverse children for starting school and literacy instruction.

Focusing on a sample of linguistically diverse learners, the specific research questions that this study seeks to answer are as follows:

(1) Can a new early literacy program in Luxembourgish that is implemented by classroom teachers using a whole class teaching procedure over a 12-week period in the last year of kindergarten improve children’s phonological awareness and letter-knowledge skills in Luxembourgish?
(2) Will possible intervention effects on these foundational skills in Luxembourgish transfer to phonological awareness, reading and spelling in German in Grade 1?
(3) How do children with low oral language proficiency in Luxembourgish respond to the intervention in Luxembourgish?

We predicted that children who received the intervention would show better performance on measures of phonological awareness and letter-knowledge in Luxembourgish than children in the “business as usual” control group immediately following the intervention in kindergarten. Furthermore, we expected that those gains would transfer to phonological awareness and literacy skills in German in Grade 1 of primary school. Finally, we hypothesized that the new program would also promote phonological awareness and letter knowledge skills in a subgroup of children with limited oral language proficiency in Luxembourgish, which would lead to more successful development of reading and spelling skills in German as compared to controls.

## Materials and Methods

The design of the study and analytical approach followed What Works Clearinghouse guidelines for credible evidence of program effectiveness (U.S. Department of Education, 2017). To achieve a good balance between internal and external validity, the study employed a two-group quasi-experimental design with an intervention group and a “business as usual” control group. It was conducted in 28 classes from eight kindergartens in Luxembourg. Data were collected in both groups contemporaneously using the same assessments. Children were followed over two academic years (from kindergarten to Grade 1 of primary school) and assessed before the start of the intervention (pretest, *t*1, kindergarten), immediately following the 12-week intervention (post-test, *t*2, kindergarten) and at delayed follow-up nine months after the intervention had ceased (*t*3, Grade 1). Groups were balanced with respect to important covariates (e.g., socio-economic status, language background, verbal abilities, non-verbal abilities, and gender). The study collected primary and secondary outcome data that were directly relevant to assess intervention effects.

A waiting list control group design was not possible because the intervention was implemented during the last year of kindergarten. Children in the control group received the standard national curriculum (described in section “Instruction Delivered to the Control Group”), which has been developed by the Luxembourg Ministry of Education (Ministère de l’Éducation nationale et de la Formation professionnelle, 2011). The study has received ethical approval by the Ethics Review Panel (ERP) of the University of Luxembourg (16-014 LITMUL CV/vg) and was notified (*notification préalable*) to the Luxembourg National Commission for Data Protection (CNPD). The study was authorized by: the *Ministère de l’Éducation nationale*, *de l’Enfance et de la Jeunesse*, the local municipal councils, the schools administrative district directors, the kindergarten coordinators and the class teachers. Informed consent was obtained from parents for all the assessment phases.

### Participants

Nine public kindergartens from villages and towns in the Center and the North regions of Luxembourg were invited to be part of the study, of which eight participated. The ninth school had to be excluded due to not agreeing to be allocated to either the intervention or the control condition– a prerequisite given the study was implemented at the school level. Schools were selected to be similar in terms of infrastructure, socioeconomic status (SES), teaching method, teacher student ratio and percentage of second language learners. Schools were paired on important confounding factors (school size, number of second language learners, and SES) and one school out of each pair was allocated to the intervention or the control group. All the classes and teachers (14 in the intervention and 14 in the control group) from the selected schools took part in the study. All the children in the second year of kindergarten were eligible and invited to participate in the study (*N* = 201). The final sample comprised 189 children (*M* = 104, *F* = 85) from 28 classrooms (*M*_age_ = 5;8 at pretest). A power calculation had indicated that with 172 children, the study would have 95% power to detect a difference between groups equivalent to Cohen’s *d* = 0.39 (*p* < 0.05, two-tailed). For 41% of the children in the sample Luxembourgish was not the dominant home language. The largest group of the Luxembourgish second language learners were Portuguese-speakers (42%), followed by French (22%) and Cape Verdean Creole-speakers (5%). Subgroup analyses were performed on 63 children with low oral language proficiency in Luxembourgish. Of those children, 79% did speak a language other than Luxembourgish at home (38% were Portuguese speakers, 17% were French-speakers and 6% were Cape Verdean Creole speakers). As a group, children with low Luxembourgish oral language proficiency came from significantly lower SES backgrounds (*M*_ISEI_ = 42.28, *SD* = 20.63) compared to the rest of the children (*M*_ISEI_ = 60.77, *SD* = 20.66), *F*(173) = 31.62, *p* < 0.001, *d* = 0.90.

Groups did not present significant pre-test differences on relevant sociodemographic factors, including age, gender, and family SES (Table 1). Baseline equivalence of the intervention and the control group was further explored on pre-specified measures (Table 2). There were no significant pre-test differences between groups on early decoding and numerical skills, vocabulary knowledge in Luxembourgish, and non-verbal reasoning. Notably, there was a significant group difference in German vocabulary at *t*3, for the entire sample (but not for the low oral language group) with children in the control group performing significantly better than children in the intervention group, *F*(1,170) = 5.42, *p* = 0.021, *d* = 0.36.

**TABLE 1:** Characteristics of schools, classes, and children in each study condition.

**Characteristics**	**Intervention group**	**Control group**
	**Mean (*SD*)**	**Mean (*SD*)**
**School-level characteristics**		
Number of schools	4	4
Number of students	22.25 (6.95)	25.00 (10.42)
Socioeconomic status^1^	4	5
**Class-level characteristics (K2)**		
Number of students	6.79 (0.98)	7.57 (1.45)
Number of classes (and teachers)	14	14
Teachers, years of teaching	18.54 (9.58)	17.42 (13.85)
Teachers, gender (female, %)	100%	100%
**Child-level characteristics, entire sample (*N* = 189)**		
Socioeconomic status (ISEI)^2^	53.44 (23.02)	55.31 (21.91)
Gender (female, %)	45%	44%
Second language learners (%)^3^	46%	38%
**Child-level characteristics, subgroup low oral language (*n* = 63)**		
Socioeconomic status (ISEI)^2^	41.67 (21.09)	42.90 (20.49)
Gender (female, %)	54%	44%
Second language learners (%)^3^	84%	75%

*K2 = second year of kindergarten; ^1^average ISEI of municipality: 1 = 35 ≤ 40, 2 = 40 ≤ 45, 3 = 45 ≤ 50, 4 = 50 ≤ 55, 5 = 55 ≤ 60, 6 = 60 ≤ 65; ^2^ISEI, International Socio-Economic Index of occupational status; ^3^second language learners are defined here as children for whom Luxembourgish was not the dominant home language.*

**TABLE 2:** Mean raw scores (*SD*) for the 12-weeks Early Literacy Intervention group and the Control Group for the control measures pre-intervention (*t*1), immediately post-intervention (*t*2), and at delayed follow-up (*t*3, with effect sizes) for the entire sample (*N* = 189) and the subgroup of children with low oral language skills (*n* = 63).

		**Entire sample (*N* = 189)**					**Subgroups low oral language (*n* = 63)**				
		
	**Reliability**	**Intervention group (*n* = 89)**		**Control group (*n* = 100)**		**Effect size**	**Intervention group (*n* = 31)**		**Control group (*n* = 32)**		**Effect size**
		***M***	***SD***	***M***	***SD***		***M***	***SD***	***M***	***SD***	
**Age (months)**											
• *t*1		67.85	3.60	68.27	3.99		67.07	3.87	67.66	3.93	
• *t*2		74.09	3.44	75.06	3.90		73.37	3.72	74.56	3.89	
• *t*3		81.31	4.25	82.35	3.99		81.14	3.79	81.92	3.88	
**Early decoding**											
• *t*1 (12)	0.95	0.27_F_	1.38	0.74_F_	2.33		0.10_F_	0.59	0.06_F_	0.35	
**Vocabulary Luxembourgish (CLT)**											
• *t*1 (40)	0.79	33.10	4.49	33.94	4.48	−0.19^2^	28.23	3.80	28.75	3.39	−0.14^2^
**Vocabulary Luxembourgish (PPVT)**											
• *t*1 (40)	0.86	29.15	6.44	29.15	6.40	0.00^2^	22.13	4.72	21.78	3.81	0.08^2^
• *t*2 (40)	0.82	31.56	5.04	31.22	5.44	0.07^2^	26.60	3.77	25.44	4.17	0.29^2^
• *t*3 (40)	0.79	33.20	4.27	33.89	4.23	−0.19^2^	28.71	3.40	29.32	3.65	−0.17^2^
**Vocabulary German (PPVT)**											
• *t*3 (228)	0.98	94.91	27.33	104.99	29.20	−0.36^2^	73.93	14.29	76.24	18.13	−0.23^2^
**Early numerical competency**											
• *t*1 (16)	0.93	2.79	3.13	3.55	4.05	−0.20^2^	1.45_F_	1.15	1.53_F_	1.52	−0.06^2^
• *t*2 (16)	0.94	4.53	4.26	5.93	4.88	−0.18^1^	2.83	2.91	3.53	3.46	−0.19^1^
**Non-verbal reasoning (WPPSI)**											
• *t*1 (29)	0.80	14.78	3.63	14.43	4.65	0.11^2^	13.61	3.60	12.03	4.47	0.39^2^

*(), maximum possible raw score; CLT, Cross Linguistic Lexical Tasks; PPVT, Peabody Picture Vocabulary Test; F, floor effect. Cohen’s d: 1 = difference in progress between groups divided by pooled initial SD; 2 = difference in means at pre-test/post-test/follow-up divided by pooled SD at pre-test/post-test/follow-up (pre-test scores were at floor/not available for same measure so could not be used). A positive effect (Cohen’s d) means that the Intervention Group did better, whereas a negative effect means results in favor of the Control Group. Reliability coefficient = Cronbach’s alpha.*

### Measures

Intervention effects were explored for skills directly taught in Luxembourgish during the intervention and for transfer and generalization of those skills to German. Primary outcome measures tapped phonological awareness and letter-knowledge in Luxembourgish. Phonological awareness and literacy measures in German were secondary outcome measures. Phonological awareness and letter-knowledge in Luxembourgish were assessed at each time point. German measures could only be administered at *t*3, after children had been exposed to German for approximately five months (see Table 3 for details of constructs assessed at each time point). To explore whether intervention effects were specific, the study also included an early numerical competency measure that was administered at *t*1 and *t*2.

**TABLE 3:** Measures and constructs assessed at each time point.

**Measures**	**Luxembourgish**	**German**
**Phonological awareness**		
**Syllable segmentation**		
*t*1	√	–
*t*2	√	–
*t*3	–	–
**Rhyme identification**		
*t*1	√	–
*t*2	√	–
*t*3	–	–
**Onset-rhyme blending**		
*t*1	√	–
*t*2	√	–
*t*3	–	–
**Onset identification**		
*t*1	√	–
*t*2	√	–
*t*3	–	–
**Onset/phoneme manipulation**		
*t*1	√	–
*t*2	√	–
*t*3	√	√
**Phoneme blending**		
*t*1	√	–
*t*2	√	–
*t*3	√	√
**Phoneme segmentation**		
*t*1	–	–
*t*2	√	–
*t*3	√	√
**Letter-knowledge**		
*t*1	√	–
*t*2	√	–
*t*3	√	–
**Reading**		
**Single word/pseudoword reading**		
*t*3	–	√
**Word-level reading comprehension**		
*t*3	–	√
**Spelling**		
*t*3	–	√
**Control measures**		
**Early decoding**		
*t*1	√	–
**Vocabulary**		
*t*1	√	–
*t*2	√	–
*t*3	√	√
**Early numerical competency**		
*t*1	√	–
*t*2	√	–
**Non-verbal reasoning**		
*t*1	√	–

No standardized language and literacy tests exist for Luxembourg. Measures in Luxembourgish were either adapted from existing German or English tests or developed for the purpose of this study. For German, standardized tests of oral language and reading were used (except for phoneme segmentation that was assessed with a newly created measure). As no norms are available for Luxembourg, raw scores were used in all analyses. Reliability coefficients were computed for all measures using the entire sample. The reliability coefficients for all the measures ranged from acceptable (>0.70) to good (>0.80) (Tables 2, 4).

**TABLE 4:** Mean raw scores (*SD*) for the 12-weeks Early Literacy Intervention group and the Control Group for the primary and secondary outcome measures pre-intervention (*t*1), immediately post-intervention (*t*2), and at delayed follow-up (*t*3, with effect sizes for intervention effects) for the entire sample (*N* = 189) and the subgroup of children with low oral language (*n* = 63).

		**Entire sample (*N* = 189)**					**Subgroups low oral language (*n* = 63)**				
		
	**Reliability**	**Intervention group (*n* = 89)**		**Control group (*n* = 100)**		**Effect size**	**Intervention group (*n* = 31)**		**Control group (*n* = 32)**		**Effect size**
		***M***	***SD***	***M***	***SD***		***M***	***SD***	***M***	***SD***	
**Primary outcomes (Luxembourgish)**											
Rhyme identification											
• *t*1 (12)	0.90	5.72	4.11	7.40	3.91		3.61	3.35	4.63	3.59	
• *t*2 (12)	0.89	8.35	3.46	8.98	3.29	0.26^1^	6.07	3.67	6.19	3.79	0.26^1^
Onset rhyme blending											
• *t*1 (12)	0.91	3.70	3.73	5.00	4.10		2.32_F_	3.27	2.84_F_	3.41	
• *t*2 (12)	0.91	9.19	3.48	6.99	3.89	0.97^1^	8.20	3.38	4.38	3.97	1.30^2^
Onset identification											
• *t*1 (12)	0.79	4.26	3.07	5.30	4.25		3.10	2.47	3.03	2.52	
• *t*2 (12)	0.83	7.45	3.52	5.99	3.34	0.79^1^	6.17	3.74	4.13	3.04	0.79^1^
Onset/phoneme manipulation											
• *t*1 (12)	0.93	0.36_*F*_	1.50	0.50_F_	1.85		0.00_F_	0.00	0.00_F_	0.00	
• *t*2 (12)	0.93	2.06_*F*_	3.34	1.30_F_	2.85		1.00_F_	2.21	0.06_F_	0.25	
• *t*3 (12)	0.91	5.25	4.26	4.86	4.11	0.13^2^	3.96	4.07	3.04_F_	3.55	0.24^2^
Phoneme blending											
• *t*1 (12)	0.92	0.65_*F*_	1.50	1.86_F_	3.25		0.29_F_	0.90	1.06_F_	2.46	
• *t*2 (12)	0.90	4.64	3.58	2.77_F_	3.29	0.90^2^	3.20	3.23	1.34_F_	2.47	0.92^2^
• *t*3 (12)	0.81	8.96	2.71	7.89	3.24	0.76^2^	8.39	2.47	6.34	3.76	0.89^2^
Phoneme segmentation											
• *t*2 (12)	0.91	3.72	3.78	1.65_F_	2.71	0.63^2^	2.37_F_	3.03	0.63_F_	1.91	
• *t*3 (12)	0.87	8.33	3.39	7.11	3.67	0.35^2^	7.32	3.86	5.96	3.70	0.36^2^
Letter-knowledge											
• *t*1 (20)	0.95	5.84	5.76	7.85	6.78		4.26	4.80	3.63	5.03	
• *t*2 (20)	0.95	15.53	5.47	9.90	6.59	1.21^1^	13.77	5.81	6.22	5.48	1.41^1^
• *t*3 (20)	0.79	19.67_*F*_	0.91	19.17_C_	1.85		19.43_C_	1.32	18.36_C_	2.72	
**Secondary outcomes (German)**											
Onset/phoneme manipulation											
• *t*3 (12)	0.91	6.20	3.84	5.98	4.26	0.05^2^	4.11	2.87	3.08	3.86	0.30^2^
Phoneme blending											
• *t*3 (12)	0.85	9.83	2.44	9.12	3.09	0.25^2^	8.96	2.83	7.12	3.63	0.57^2^
Phoneme segmentation											
• *t*3 (12)	0.84	8.99	2.73	7.65	3.12	0.46^2^	7.96	3.19	5.88	3.15	0.66^2^
Single word reading											
• *t*3 (72)	0.98	8.62	9.39	9.47	9.73	−0.09^2^	6.14	6.73	6.16	6.59	0.00
Pseudoword reading											
• *t*3 (72)	0.96	13.75	8.05	14.97	7.98	−0.15^2^	11.71	7.63	12.80	7.01	−0.15^2^
Word reading comprehension											
• *t*3 (72)	0.95	12.73	5.02	11.86	5.61	0.16^2^	11.04	4.63	9.40	3.81	0.39^2^
Spelling											
• *t*3 (40)	0.93	34.25	3.24	32.37	7.50	0.32^2^	33.14	3.88	28.60	10.66	0.57^2^

*(), maximum possible raw score; C, ceiling effect; F, floor effect. Cohen’s d: 1 = difference in progress between groups divided by pooled initial SD; 2 = difference in means at post-test/follow-up divided by pooled SD at post-test/follow-up (pre-test scores were at floor/not available for same measure so could not be used). A positive effect (Cohen’s d) means that the Intervention Group did better, whereas a negative effect means results in favor of the Control Group. Reliability coefficient = Cronbach’s alpha.*

#### Phonological Awareness in Luxembourgish

A new test in Luxembourgish was developed based on the German *Test for Phonological Awareness Skills* (TPB, *Test für Phonologische Bewusstheitsfähigkeiten*, Fricke and Schäfer, 2011). The test contains seven tasks that cover a range of linguistic unit-sizes and degrees of explicitness. Each task contains 12 test items and three practice items. The following tasks were administered: (1) *syllable segmentation* (segment a spoken word into its constituent syllables); (2) *rhyme identification* (identify the word that rhymes with a target among a choice of three); (3) *onset-rhyme blending* (pronounce a word by blending onset and rhyme that are spoken with a pause of one sec. in between); (4) *onset identification* (identify the word that has the same onset as a target among a choice of three); (5) *onset/phoneme manipulation* (say a non-word by deleting the initial onset or phoneme of a real word); (6) *phoneme blending* (pronounce a word by blending phonemes that are spoken with a pause of one sec in between); (7) *phoneme segmentation* (segment a word into its constituent phonemes). Two composite scores were created using Principal Component Analysis (PCA): A large unit size component (based on rhyme identification, onset-rhyme blending, and onset identification) and a small unit size component (based on onset/phoneme manipulation, phoneme blending at *t*1 and on onset/phoneme manipulation, phoneme blending, and phoneme segmentation at *t*2 and *t*3). Component loadings were all above 0.60 and standardized component scores were used in the subsequent analyses.

#### Phoneme Awareness in German

Children completed the *phoneme blending* and the *onset/phoneme manipulation* tasks from the German standardized *Test for Phonological Awareness Skills* (TPB, Fricke and Schäfer, 2011). *Phoneme segmentation* was assessed with a newly developed task. For all the phonological awareness tasks, task design and administration/scoring procedures were identical as for the Luxembourgish equivalent described above with the exception that words were in German. A standardized component score was created from the three tasks (loadings were above 0.60) and used in the analysis.

#### Letter-Knowledge

Children were asked to say the sound or the letter name of 20 single letters that were presented on a computer screen in upper- and lower-case characters.

#### Reading

The *pseudoword reading* and the *single word reading* subtests from the standardized *Salzburg Reading and Spelling Test* (SLRT-II, *Salzburger Lese und Rechtschreibetest*, Moll and Landerl, 2010) were administered. Children have to read aloud German words or pseudowords as fast as possible in one min. The scoring procedure was according to the manual, which considers an item as correct if it is read correctly in German. A standardized component score was created from the two tasks (loadings were above 0.60) and used in the analysis as a measure of single word/pseudoword reading in German. Children also completed the *word comprehension* subtest from the standardized *Reading Comprehension Test for First- to Sixth-Graders* (ELFE 1-6, *Ein Leseverständnistest für Erst- bis Sechstklässler*, Lenhard and Schneider, 2006). Test items are composed of a picture and four words presented in a column, one of which is the correct word for the picture. Children have to silently read the words and underline the word that matches the picture. The test stops after three min.

#### Spelling in German

Children completed the *Hamburg Writing Test* for Grade 1 (HSP-1+, *Hamburger Schreibprobe*, May, 2002) that assesses orthographic knowledge in German. Children are asked to write four single words that are individually dictated to them and a sentence.

#### Early Decoding

Children completed an *early decoding test* that was developed for the purpose of this study and administered in Luxembourgish (Wealer, 2019). Children have to read aloud 12 non-words that are presented on two A4 sheets.

#### Vocabulary in Luxembourgish and German

Children completed two versions of the *Peabody Picture Vocabulary Test* (PPVT-4, Dunn and Dunn, 2007); one Luxembourgish version specifically created for the purpose of research projects conducted in Luxembourg and the adapted standardized German version of PPVT (Lenhard et al., 2015). Children have to identify a target picture out of a choice of four to match a spoken word. The German test was administered according to the manual. As no norms or items statistics are available for the Luxembourgish version, a predetermined fixed set of 40 items was administered to all children. In addition, children completed an experimental, receptive vocabulary test (Cross-linguistic Lexical Tasks, CLT, Haman et al., 2017) that contains early acquired words in Luxembourgish. Children had to match a spoken word to a picture out of a choice of four.

#### Early Numerical Competency

Children completed a number naming task in which they were asked to name 16 numbers between four and 100.

#### Non-verbal Reasoning

The *matrix reasoning subtest* of the *Wechsler Kindergarten and Primary Scale of Intelligence* (WPPSI III, Wechsler, 2007) was administered in which children have to complete figures by finding the missing piece among four or five possible drawings.

#### Questionnaires

Self-completion questionnaires were designed for the purpose of this study and administered to parents (sent and returned in sealed envelopes via teachers) and teachers (handed out and collected in sealed envelopes). Parent questionnaires were collected during two periods: enrollment (for all children) and at delayed follow-up (only for the children in the intervention group). All parental data collection instruments were developed in Luxembourgish, German, French, and Portuguese (the most frequent languages spoken in Luxembourg) as well as in English. To keep enrollment materials brief, the *initial background questionnaire* was short (two-pages) and contained information about the child’s age, nationality, year of entry into Luxembourg, developmental and educational history. The questionnaire also contained questions about the language usage in the home and parents’ language background, their education and occupation. The responses to the open-ended occupational questions were coded to four-digit ISCO codes and transformed into the International Socio-Economic Index of occupational status (ISEI, Ganzeboom and Treiman, 1996). Included in the two-page *parent post-intervention questionnaires* were items that asked about the perception of the effectiveness of the intervention, the home use of the materials, and the child’s responsiveness to the intervention. It also contained open-ended questions where parents could express how they felt about the intervention and how they were interacting with the materials at home. To reduce social desirability bias, the questionnaire was anonymous. A two-page *teacher practice questionnaire* providing information on years of teaching experience and type and frequency of activities used in the class to promote early literacy skills development was completed by all teachers. In addition, teachers in the intervention group were invited to complete an anonymous four-page *teacher post-intervention questionnaire* providing quantitative and qualitative data. The questionnaire contained a balanced mix of closed and open-ended questions in relation to the acceptability of the intervention, including satisfaction and perception of and experience with the intervention and encountered challenges and suggestions.

### Procedure

At *t*1 and *t*2, each child was tested individually in two sessions and at *t*3 in three sessions of 20–30 min each, in a quiet area of the school. Tests were grouped by language of administration and administered in a fixed sequence by one of the authors (CW) and trained research assistants. Test administrators were native- or native-like speakers of the respective test language.

### The Intervention Program

Children in the intervention group received the LALA *Lauter lëschteg Lauter* (many funny sounds) program which aims to improve children’s phonological awareness skills, develop their letter-knowledge, promote print and book awareness, and increase literacy engagement. The intervention runs over 12 weeks in kindergarten. It is delivered by the classroom teacher to all Year 2 kindergarten students (groups ranging from 5 to 9 children) in a class. The intervention combines direct instruction with a pedagogical approach aligned with playful learning. It uses multimodal techniques that foster the development of children’s early literacy skill in a structured and systematic way embedded in language-rich and meaning-oriented activities. The intervention is embedded in an engaging storyline that evolves around the hand puppet macaw Lala who visits children from Brazil to discover the sounds of Luxembourgish. All activities are carried out with the support of the hand puppet to increase child motivation and engage their imagination. Playful learning stands at the heart of the program and all activities. The aim was to combine children’s sense of curiosity with structured learning activities. Children engage in fun activities with the Lala character including: discovering sounds and letters in words (in Luxembourgish and other languages or pseudowords); learning the sounds of letters with the help of mnemonics (e.g., songs, flashcards, and movement); blending individual sounds into real words (e.g., “parrot talk” activity), feeling and experiencing letters using divers materials (e.g., writing letters in sand). The intervention also places emphasis on developing children’s reading engagement and book awareness through lively and age appropriate stories that are matched to children’s sound and letter knowledge. A brief overview of the program can be seen in this video: https://cutt.ly/LALA-Program and some of the resources used in the program (e.g., flashcards, stories, and songs) can be accessed here: https://cutt.ly/LUMI.

The intervention contains 48 teaching units of 25 min each and is administered four times per week (total intervention time: 20h). During the first three units of each week new content is introduced that is consistently consolidated in the fourth weekly session. In the first two weeks (units 1–9), children engage in phonological awareness activities at the syllable, rhyme and onset-rhyme level. Weeks 3–12 (units 10–48) focus on training phoneme awareness and the linkage between phonemes and their letters. The phoneme awareness activities include phoneme identification (weeks 3–12), phoneme blending (weeks 4–12), and phoneme segmentation (weeks 9–12). In total, 23 phonemes and their respective letters are introduced at the rate of three phonemes/letters per week (one phoneme-letter combination per unit). The choice and sequence of introducing the phonemes/letters was based on the following criteria: sound-letter correspondences that Luxembourgish shares with German; only one sound per letter; frequent and salient sounds are introduced first; auditory similar sounds or visually similar letters are separated in the sequence; sounds represented by more than one grapheme (two digraphs and one trigraph) are introduced last (Beck and Beck, 2013).

Another key element in the intervention is the regular storytelling and book reading activity (weeks 3–12). Twenty-three developmentally appropriate short stories were developed that incorporate the taught sounds and letters (one story for each sound-letter combination) in order to further consolidate sound-letter linkage while at the same time engaging in a meaning-oriented literacy activity and fostering print and book awareness. Teachers were provided with big books (A1 size) containing the stories. They were asked to spend time on reading aloud to their class and to draw children’s attention to the written words in the text by pointing to the print as it is read and by talking about the text and the letters in words. In addition, each child was given a smaller copy of the book (A4 size) and the corresponding flashcards. The intervention also encourages children to use emergent forms of writing such as writing letters in sand or in the air. The intervention contains two revision weeks (weeks 6 and 12) during which children receive further opportunities to consolidate previously learned content. In addition to the revision weeks and the weekly consolidation sessions, revision of previously learned content is also regularly incorporated throughout the intervention. Each sound is, for example, revised systematically at least seven times in the context of phoneme awareness activities after its initial introduction. Teachers can adjust the difficulty level of most activities to match the skill level of individual students.

The intervention follows established strategies for working with linguistically diverse learners based on previous research (e.g., August et al., 2005; Chumak-Horbatsch, 2012; Richards-Tutor et al., 2016) and professional opinions of practitioners. In addition to culturally appropriate materials, the approach includes: taking advantage of children’s first language; clarifying meanings of basic words; modulating language demands; gestures and visual support; creating an inclusive learning environment; valuing the unique linguistic and cultural background of each learner; providing ample opportunities for practice and revision; using systematic and explicit instructional routines and building on children’s familiar experiences.

A goal was also to increase print access and literacy engagement. Toward this end, resources and materials were made available to parents to encourage their involvement. A trilingual parent guide was developed in Luxembourgish, French, and Portuguese (the most frequent languages spoken in Luxembourg) with easily understandable practices and specific strategies that can be implemented from home to support children’s literacy development. Books and other materials were given to children and recommendations for parents were developed on how to effectively use those resources at home, irrespective of the home language.

### Teacher Training and Support

The 14 teachers in the intervention group were trained during one day (8 h) in a single session by members of the research team (PE and CW). A refresher training session of 2h was held in smaller groups, within each school, in the week prior to the start of the intervention. Teacher training centered around describing the intervention including its rationale, intervention procedure and activities, the importance of using rich language, how to effectively use puppetry as a teaching tool, and strategies on how to effectively support children from linguistically diverse backgrounds. Teachers also received a detailed scripted manual describing the activities together with all the necessary material. The main role of the research team was to provide teacher support as well as monitor treatment fidelity and attendance through observations and tutorials. Following these, feedback was provided and questions were addressed as necessary.

### Instruction Delivered to the Control Group

Children in the control group received the national kindergarten curriculum that is uniform across public schools in Luxembourg. Study content and levels of competences to be acquired are fixed in the Grand-ducal Regulation of August 11, 2011 (Ministère de l’Éducation nationale et de la Formation professionnelle, 2011). Play-based learning in a holistic approach is the dominant educational approach and the curriculum is relatively broadly defined. Oral language development in Luxembourgish forms a major part of kindergarten instruction. Children acquire these skills through social interactions and play-based learning activities. With reference to the acquisition of early literacy skills, at the end of kindergarten, children are expected to be able to: identify rhymes and initial sounds and segment words; differentiate different written signs; handle a book; discover the social use of writing; discover their first name among other names; recognize well-known pictograms; follow the course of events in an easy text that is read to them. Kindergarten activities should not focus on explicit acquisition of skills but instead rely on a global, holistic approach by immersing children in familiar and stimulating contexts (Ministère de l’Éducation nationale et de la Formation professionnelle, 2011). Direct pre-literacy instruction including more formal letter teaching should only be implemented incidentally if children manifest readiness signs.

Teachers in the control group indicated that they would engage in activities that foster phonological awareness approximately two to three times a week, mostly at the level of the rhyme and syllable. Letters were not systematically introduced. Generally, teachers in the control group indicated that they would work with letters if children explicitly asked for it. Big book activites were not part of the classroom activities of the control group.

### Fidelity of Implementation

Each teacher in the intervention group was observed four times on-site, delivering a unit to the class by members of the research team (PE and CW). Adherence to the intervention manual was graded on a 5-point scale (1: *several aspects missing*, 2: *some aspects missing*, 3: *according to manual*, 4: *according to manual with good use of resources/strategies*, 5: *according to manual with very good use of resources/strategies*). In addition, children’s engagement was rated on a 5-point scale (1: *poor responsiveness*, 2: *below expected responsiveness*, 3: *expected responsiveness*, 4: *above expected responsiveness*, 5: *extraordinary responsiveness*). Teachers also completed a self-report register for each unit, including attendance rates of children. Members of the research team (PE and CW) held monthly school tutorials with teachers. Children were also asked to rate their satisfaction with the intervention on a rating scale from one to three (1: *did not like it*, 2: *liked it*, 3: *liked it very much*). At the end of the intervention, a focus group was held with 11 out of the 14 intervention teachers.

## Results

Data are reported on 89 children (*M*_age_ = 5;8 at *t*1) in the early literacy intervention and 100 children (*M*_age_ = 5;8 at *t*1) in the control group. In total, 17 participants (9%, *n* = 8 from intervention; *n* = 9 from control group) could not be followed up at delayed post-test (*t*3) because they had either moved to another country or to a school district in Luxembourg for which no study consent had been obtained. We classified children as presenting low oral language proficiency in Luxembourgish if they scored in the lowest tertile on a Luxembourgish vocabulary composite score at *t*1 computed via PCA combining the Luxembourgish PPVT and the CLT measures (*n* = 63). Out of the 63 children in the low oral language group, 31 children (*M*_age_ = 5;7 at *t*1) were in the intervention group and 32 children (*M*_age_ = 5;8 at *t*1) in the control group.

All analyses were performed on an intention-to-treat basis (Gupta, 2011) in SPSS version 24.0 (IBM Corp, 2016). Analyses were conducted twice, once on the entire sample (*N* = 189) and once on the children in the low oral language group (*n* = 63). Data (component scores when available) were analyzed using analyses of covariance (ANCOVA) controlling for baseline performance on each variable (the autoregressor) whenever available (U.S. Department of Education, 2017). ANCOVA was also used when groups differed significantly on any of the control variables (see above regarding German vocabulary). Statistical analyses including measures that tapped into abilities that could not be assessed at *t*1 were performed using ANOVAs. To check for homogeneity of regression slopes, an interaction term between group and pre-test score measures was initially included in all the models. With the exception of letter-knowledge at post-test, the interaction term was not significant for any measure, so it was subsequently dropped from the models. Little’s MCAR test confirmed that missing data could be considered to be missing completely at random, χ^2^ = 94.72, *df* = 75, *p* = 0.062.

All 14 teachers delivered 48/48 units and children completed on average 46.24/48 (*SD* = 2.53, range 36–48) teaching units or 96% of the intervention. There was no significant relationship between the number of teaching units attended and the degree of improvement on any of the outcome measures. Children received an average rating of responsiveness to the intervention of 3.01/5 (*SD* = 0.47, range: 1.75–3.75). Teachers achieved a mean adherence to program quality rating of 3.12/5 (*SD* = 0.47, range: 2.50–3.80) indicating that the intervention was delivered as intended. There was no significant group difference on number naming at *t*2 (after controlling for *t*1 group differences), *F*(1,182) = 1.70, *p* = 0.194, *d* = −0.19 (Table 2).

Descriptive statistics and effect sizes for all primary and secondary outcome measures at pre-test (*t*1), immediate post-test (*t*2) and delayed follow-up-test (*t*3) for the intervention and the control groups are shown in Table 4. As expected, the data exhibited floor and ceiling effects on a number of measures. As such effects in data analysis can lead to biased estimates and distorted significance testing, effect sizes and significance tests were not computed on those measures (Wang et al., 2009). Children scored at ceiling on syllable segmentation in Luxembourgish at *t*1, results on this measure were therefore excluded. Data inspection showed that distributions on letter-knowledge at *t*2, and the German phonological awareness and literacy measures at *t*3 were skewed and these measures also presented an extreme outlier. Analyses including these measures were therefore performed using square root (for moderate skewed data: phonological awareness, letter-knowledge, word reading) or log transformations (for substantial skewed data: spelling) for normality (Tabachnick and Fidell, 2012). To ease interpretation and provide raw data, the tabled values are untransformed.

### Effects of Intervention for the Entire Sample

Controlling for *t*1 scores, large effects in favor of the intervention group were observed on phonological awareness in Luxembourgish compared to the controls immediately post intervention: large unit size, *F*(1,182) = 53.40, *p* < 0.001, *d* = 1.09; small unit size, *F*(1,182) = 36.62, *p* < 0.001, *d* = 0.90. The effect was maintained at delayed follow-up: small unit size, *F*(1,169) = 9.58, *p* = 0.002, *d* = 0.48. Large post-test effects in favor of the intervention group also emerged on letter-knowledge after controlling for *t*1 group differences: *F*(1,182) = 145.86, *p* < 0.001, *d* = 1.78. At delayed follow-up, scores on the letter-knowledge task presented a ceiling effect.

We also explored the extent to which the intervention in Luxembourgish produced transfer effects on phonological awareness and literacy measures in German at delayed follow-up. As lexical knowledge can affect performance on literacy tasks, analyses including the German literacy measures were conducted controlling for German vocabulary. Children in the intervention group performed significantly better than children in the control group on phonological awareness, *F*(1,169) = 10.96, *p* = 0.001, *d* = 0.51, word-level reading comprehension, *F*(1,169) = 4.01, *p* = 0.047, *d* = 0.31, and spelling, *F*(1,169) = 6.65, *p* = 0.011, *d* = 0.40. No significant group difference emerged on single word/pseudoword reading, *F*(1,169) = 0.02, *p* = 0.890, *d* = 0.00.

### Effects of Intervention for Children With Low Oral Language Proficiency in Luxembourgish

Given that the smaller sample size of the subgroup analysis (*n* = 63) leads to a reduction in power, measures of effect size are important to take into account to assess intervention effects. The results showed large and significant effects in favor of the intervention group immediately post-intervention (after controlling for *t*1 group differences) on Luxembourgish phonological awareness-large unit, *F*(1,59) = 16.03, *p* < 0.001, *d* = 1.04, Luxembourgish phonological awareness-small unit, *F*(1,59) = 17.54, *p* < 0.001, *d* = 1.09, and letter-knowledge, *F*(1,59) = 44.67, *p* < 0.001, *d* = 1.74. Effects on Luxembourgish phonological awareness-small unit were maintained at delayed follow up with a medium effect size, *F*(1,50) = 5.95, *p* = 0.018, *d* = 0.69. Significant medium effects also emerged at delayed follow-up on German phonological awareness, *F*(1,51) = 5.57, *p* = 0.022, *d* = 0.66, with children in the intervention group outperforming children in the control group. As for the entire sample, analyses on the German literacy measures at *t*3 were conducted controlling for German vocabulary and showed that children in the intervention group performed significantly better than children in the control group in spelling, *F*(1,50) = 5.38, *p* = 0.024, *d* = 0.66. This group difference can be considered medium in magnitude. On word-level reading comprehension, children in the intervention group outperformed children in the control group but the effect size was small (*d* = 0.34) and did not reach statistical significance, *F*(1,50) = 1.42, *p* = 0.239. No significant group difference emerged on single word/pseudoword reading in German, *F*(1,50) = 0.02, *p* = 0.960, *d* = 0.00.

### Acceptability of the Intervention

The post-intervention teacher questionnaire was completed by all 14 teachers in the intervention group. Results showed that 100% of the teachers were satisfied with the intervention, i.e., they enjoyed working with the program; would like to continue using it in the following year; judged that the intervention was appropriate in addressing the problem and was suitable to the Luxembourgish kindergarten context; reported feeling better prepared to identify their pupils strengths and weaknesses and perceived the intervention as effective. Teachers also expressed the view that they felt that the systematic and explicit approach was important and that their children enjoyed working with the program. Their main struggle was to incorporate the activities four times per week. They also recurrently expressed the wish to be able to use the intervention with children who did not show progress as the others in smaller groups or individual teaching sessions. Data analyses of the post-intervention parent questionnaire showed that 70% of the parents (*n* = 57) returned the questionnaire which can be considered a very good response rate (Mangione, 1995). In general, parents were most satisfied with the intervention: 98% of the children were reported to speak about the intervention at home; 95% of the parents indicated that their child had learned new things through the intervention and frequently used the resources at home; and 98% of the parents indicated that the parental material was adequate and easy to understand. The recurrent themes that emerged from the open-ended questions were: increased interest in letters and print; joy and enthusiasm of learning with the program; perception that the intervention prepared the child well for literacy acquisition in German; happiness that their child took part in the intervention and wish that the program would continue. Ninety-nine percent of the children indicated that they had enjoyed working with the intervention program at school.

## Discussion

In this study, we evaluated the effectiveness of a novel program, designed to promote foundation skills of literacy acquisition in linguistically diverse kindergarten children from Luxembourg who go on to learn to read and write in German. Program development was guided by the Simple View of Reading theoretical framework (Gough and Tunmer, 1986) and based on previous longitudinal and intervention studies suggesting a causal link between phonological awareness and letter-knowledge as prerequisites for reading and spelling across orthographies (Melby-Lervåg et al., 2012). The LALA program differs from previous intervention studies from Germany and Luxembourg, which have almost exclusively focused on exploring the effectiveness of the German training program HLL (e.g., Schneider et al., 1997, 2000; Plume and Schneider, 2004; Bodé and Content, 2011; Pfost et al., 2019) that was originally developed within a monolingual orientation (Lundberg et al., 1988). Of particular relevance to this study is that in linguistically diverse students a “traditional” phonics-based instructional approach often may require adaptations, and phonological awareness and letter-knowledge should be taught within a broad language curriculum (Stuart, 1999, 2004).

Here we took a more holistic and contextualized approach than other interventions. Activities of phonological awareness and letter-knowledge were embedded in a literacy and language rich context that also stimulated print awareness and literacy engagement and created regular opportunities for book sharing and dialogic interactions. The 12-week LALA program was delivered in Luxembourgish by class teachers to whole-classes in mainstream kindergarten settings. Effects were monitored immediately post-intervention and at a 9 months delayed follow-up, approximately 5 months after children had started to learn to read and write in German. Gains in the primary outcome measures in Luxembourgish as well as transfer to German language and literacy measures were explored by comparison with children who had followed the standard kindergarten curriculum.

It was shown that after only 12 weeks of teaching in kindergarten, the children in the intervention group had significantly better phonological awareness skills in Luxembourgish than children who had not followed the program and this effect was maintained 9 months later in Grade 1. The program was also effective in enhancing children’s letter-knowledge skills. A very large effect in favor of the intervention group was observed immediately post-intervention. At delayed follow-up, both groups seemed to have mastered letter-knowledge, which is not unusual after approximately 5 months of formal literacy instruction in German. Previous studies have shown that children generally acquire letter-knowledge and decoding skills fast in German due to its relatively consistent orthography (Aro and Wimmer, 2003). While the intervention exerted significant effects on emergent literacy skills, it did not have an impact on children’s performance on an early numerical competency measure. Results on our primary outcome measures support our expectations. These findings from the Luxembourg educational context corroborate conclusions drawn from studies in other countries, languages, and orthographies and indicate that phonological awareness and letter-knowledge can be successfully trained with high quality intervention prior to formal reading instruction (Bus and van IJzendoorn, 1999; Schneider et al., 2000; Melby-Lervåg et al., 2012; Kelly et al., 2019; Pfost et al., 2019).

In line with the predictions of the Simple View of Reading, the intervention also produced improvements in literacy skills. There is evidence that the program in Luxembourgish improved phoneme awareness, word-level reading comprehension and spelling in German in Grade 1. However, no significant intervention effect was observed for German single word/pseudoword reading. This latter result is not surprising. Previous studies have shown that, in German, spelling and reading comprehension depend to a greater extend on phonological awareness skills than word-level decoding (Wimmer et al., 2000; Pfost, 2015; Fricke et al., 2016). Early literacy intervention studies from Germany also consistently report larger effects on reading comprehension and spelling than on decoding, for which effects are generally negligible (Fischer and Pfost, 2015).

The finding that effects of an intervention focusing on early-literacy skills in Luxembourgish transferred to some skills in German, i.e., phonological awareness, word-level reading comprehension and spelling, is encouraging. It is consistent with linguistic theories of language interdependence and facilitation according to which development in one language can influence the development of certain language domains in another language (e.g., Cummins, 1979; MacSwan and Rolstad, 2005; Rothman, 2011). This intervention study corroborates findings from correlational research indicating cross-linguistic transfer in phonological awareness and decoding skills (Melby-Lervåg and Lervåg, 2011). Important to note is that Luxembourgish and German are typologically close and structurally similar languages – characteristics that have been suggested to make language transfer more likely because learners may recognize common features across languages (e.g., phonological forms), which facilitates transfer of the knowledge of those features from one language to another (Connor, 1996). It remains to be seen whether a similar magnitude of cross-linguistic transfer would emerge in other language combinations and in languages that share fewer features than Luxembourgish and German.

Our findings contrast with a recent intervention study from Germany that did not find improvements in reading comprehension (using the same measure as used in the current study) after 20-weeks of training phonological awareness and letter sound knowledge in a random group of children (Pfost et al., 2019). The study did, however, identify small to medium effects on reading comprehension in children at risk of reading difficulties. In our study, German was a second language for almost all the children (98%). Learning to read in a second language is arguably harder than learning to read in a first and low proficiency in the instructional language has been identified as an important risk factors for developing literacy difficulties (Catts et al., 2012). National studies have consistently shown that by the age of nine, over 40% of Luxembourg’s student do not reach expected reading levels (Hoffmann et al., 2018). It is therefore possible that linguistically diverse children may struggle more with basic reading than monolingual learners even in a comparably consistent orthography. Thus, early interventions might show larger effects in such study populations. Our results also contrast with those of an earlier study from Luxembourg that did not find transfer effects of a phonological awareness intervention in Luxembourgish to spelling in German with a random sample of linguistically diverse children (Bodé and Content, 2011). Notably, that study used the same HLL training program as Pfost et al. (2019) but did not include activities to foster letter-sound knowledge. Several key studies suggest that interventions combining phoneme awareness with letters-knowledge training are more effective than the isolated training of phonological awareness (Hatcher et al., 1994; Bus and van IJzendoorn, 1999; Schneider et al., 2000; Bowyer-Crane et al., 2008). Furthermore, the program that was evaluated in the current study had been particularly adapted to the needs of multilingual learners. The LALA program ensured that direct instruction of code-related skills was carried out in the context of a broadly-based literacy enrichment program. The program, for example, used an inclusive pedagogical approach that encompasses taking advantage of children’s first language, ensuring understanding of basic words, culturally appropriate materials and providing ample opportunity for practice and revision.

Another encouraging finding was that a subgroup of children identified as having low oral language proficiency in Luxembourgish also demonstrated improvements in literacy following intervention. Notably 79% of these children were Luxembourgish second language learners and many came from low-income homes which is associated with a greater risk of developing literacy difficulties (Snow et al., 1998). The question whether this group of children also benefited from the intervention was important. It is possible that effects found for a random sample of kindergarten children were driven by substantial improvements of language-majority children with good skills in the language of the intervention, while children from less privileged backgrounds might not have benefited to the same degree from the intervention. Furthermore, it has been argued that cross-linguistic transfer effects are weaker in children from lower SES backgrounds than in children from middle and higher SES backgrounds because it is presumed that the latter have more decontextualized first language skills that might facilitate second language acquisition in school settings (Cummins, 1979, 2004). Our findings did not confirm this but future studies will need to investigate the interaction between SES and children’s language background in Luxembourg in more detail.

An important finding was the high acceptability of the intervention by deliverers (i.e., teachers) and recipients (i.e., children and parents). Adherence to the program and absence of intervention discontinuation, positive satisfaction ratings and high perception of effectiveness are encouraging indications of the feasibility of the intervention delivered by general education kindergarten teachers. It also provides converging evidence that it is possible to nest an early literacy intervention that relies on teacher-led learning methods within a play-based and holistic approach to learning context. Previous studies have shown that kindergarten teachers can be reluctant to implement structured phoneme analyses activities and to formally teach letter sounds (Schneider et al., 2000; Yeh, 2003; Bodé and Content, 2011). This was not the case in our study, which is encouraging. Teachers judged the intervention activities to be developmentally appropriate and they particularly appreciated its playful approach. A crucial element was that explicit and systematic instruction in phonological awareness and letter-knowledge was embedded in a language- and literacy-rich environment and therefore, not perceived by teachers as a decontextualized method of instruction. Taken together these findings suggest that an explicit and systematic approach to early literacy instruction is not incompatible with a playful pedagogy.

The study had some limitations that should be acknowledged. First, the study design was not a randomized controlled trial (RCT). As the intervention was tested in a natural setting, which is a strength in terms of external validity, random allocation into groups was not possible because of practical reasons and ethical concerns. Also, RCT study designs are not common in educational research in Luxembourg. A second limitation is that the study only allows to draw conclusions based at the whole-program level. Future research would be needed to identify specific program elements that might be particularly powerful in driving improvements in literacy skills. Another question for future studies would be to determine the quality assurance mechanisms necessary to ensure that the intervention benefits remain replicable. Despite these limitations, the positive effects identified with this quasi-experimental study suggest that it would be beneficial to adapt the LALA program into a more sustainable package and to explore its effectiveness in a larger RCT.

From an applied educational viewpoint, the results are relevant. The LALA program could be successfully delivered by regular teachers, it could be relatively easily integrated into the existing kindergarten context and did not require additional staff resources. Also, in practice, the intervention has been maintained by all the teachers from the intervention group after the end of the study and the program is now part of class routine in those schools. According to the Promising Practices Network (2014) and the What Works Clearinghouse (U.S. Department of Education, 2017) effect sizes if 0.25 or larger are considered as “substantively important.” Following this standard, the results of this study can be interpreted as educationally meaningful and of practical interest.

### Conclusion

This study provides further evidence that systematic and explicit instruction in phonological awareness and letter-knowledge, and practicing these skills in the context of a language- and literacy-rich teaching context can be an effective classroom-based approach to prepare linguistically diverse children for literacy instruction in real world circumstances. It provides important empirical evidence to better support children for second language literacy learning and represents a step into the direction of strengthening the evidence-base for prevention initiatives for all learners. To meet the global challenge of ensuring equal opportunity for all, we will clearly need to have more demonstrations of evidence-based instructional practices and support programs for use in early school settings educating linguistically, culturally, and socio-economically diverse populations.

## Data Availability Statement

The raw data supporting the conclusions of this article will be made available by the authors, according to the EU General Data Protection Regulation (GDPR), to any qualified researcher.

## Ethics Statement

The studies involving human participants were reviewed and approved by Ethics Review Panel (ERP) of the University of Luxembourg. Written informed consent to participate in this study was provided by the participants’ legal guardian/next of kin.

## Author Contributions

PE and SF contributed to conception and design of the study. PE and CW wrote the program manual. CW collected the data, organized the database, and performed the statistical analysis. All authors contributed toward the data analysis plan and the interpretation of the data. PE wrote the first draft of the manuscript. CW wrote sections of the manuscript. SF revised and refined the manuscript. All authors contributed to manuscript revision, read and approved the submitted version. CW was the Ph.D. student working on the project that forms the basis of this manuscript (Wealer, 2019) and was jointly supervised by PE and SF.

## Conflict of Interest

PE and CW declare that they are authors on the LALA program manual. PE declares that she has received a Proof of Concept grant from the Luxembourg National Research Fund, FNR (Po17/11757260) to explore the commercial, innovation or social potential of the LALA program. She is a minority shareholder of Lumi that holds the license to the LALA program.

The remaining author declares that the research was conducted in the absence of any commercial or financial relationships that could be construed as a potential conflict of interest.
